# Hypothermia is an independent risk factor for prolonged ICU stay in coronary artery bypass surgery: an observational study

**DOI:** 10.1038/s41598-023-31889-x

**Published:** 2023-03-21

**Authors:** Yi-Chia Wang, Hsing-Hao Huang, Pei-Ching Lin, Ming-Jiuh Wang, Chi-Hsiang Huang

**Affiliations:** 1 Department of Anesthesiology, National Taiwan University College of Medicine and National University Hospital, National Taiwan University Hospital, 7 Chung-Shan South Road, Taipei, Taiwan 10002; 2 National Taiwan University Cancer Center, No. 57, Ln. 155, Sec. 3, Keelung Rd., Da’an Dist., Taipei City, 106 Taiwan

**Keywords:** Outcomes research, Risk factors

## Abstract

Maintenance of normothermia is a critical perioperative issue. The warming process after hypothermia tends to increase oxygen demand, which may lead to myocardial ischemia. This study explored whether hypothermia was an independent risk factor for increased morbidity and mortality in patients receiving CABG. We conducted a retrospective observational study of CABG surgeries performed from January 2018 to June 2019. The outcomes of interest were mortality, surgical site infection rate, ventilator dependent time, intensive care unit (ICU) stay, and hospitalization duration. Data from 206 patients were analysed. Hypothermic patients were taller (p = 0.012), had lower left ventricular ejection fraction (p = 0.016), and had off-pump CABG more frequently (p = 0.04). Our analysis noted no incidence of mortality within 30 days. Hypothermia was not associated with higher surgical site infection rate or longer intubation time. After adjusting for sex, age, cardiopulmonary bypass duration, left ventricular ejection fraction, and EuroSCORE II, higher EuroSCORE II (p < 0.001; odds ratio 1.2) and hypothermia upon ICU admission (p = 0.04; odds ratio 3.8) were independent risk factors for prolonged ICU stay. In addition to EuroSCORE II, hypothermia upon ICU admission was an independent risk factor for prolonged ICU stay in patients receiving elective CABG.

## Introduction

There are many well-documented predictive instruments for operative risk assessment in adult cardiac surgery, such as EuroScore and STS score^1,2^. These preoperative assessments provide valuable information for risk stratification and clinical management. In addition to these unmodifiable risk factors, perioperative management also influences postoperative outcomes. Previous research on anaesthesiologists’ performance^3^, intravenous fluid supplement^4^, and temperature control highlights the critical role of perioperative management in coronary artery bypass grafting (CABG) surgery.

Maintaining perioperative normothermia has increasingly gained attention, especially in an era of enhanced recovery after surgery^5^. Theoretically, perioperative hypothermia could lead to complications, such as coagulopathy, increased transfusion requirement, surgical site infection (SSI), delayed drug metabolism, prolonged recovery, shivering, and thermal discomfort^6,7^. Perioperative hypothermia has been shown to increase metabolic demands during rewarming and increase the risk of postoperative myocardial ischemia^8,9^. Maintenance of normothermia has been associated with lower 30-day morbidity and mortality in orthopaedic surgeries and lung resection surgeries^10,11^, but its benefits in elective cardiac surgeries is not clear. Studies of fast-track cardiac surgeries have shown that hypothermia upon intensive care unit (ICU) admission was a risk factor for delayed extubation^12–15^. However, temperature could be taken by blood temperature, nasopharyngeal temperature, tympanic temperature, bladder temperature, or rectal temperature, which hindered comparison between studies^16,17^. It is not apparent whether hypothermia upon ICU admission is correlated with other outcomes. In a retrospective study, transient hypothermia was common after elective cardiac surgery but was not associated with hospital mortality^18^. Other studies of CABG outcomes have not included body temperature in their analyses^19,20^.

Because the warming process after hypothermia tends to increase oxygen demand, which may lead to myocardial ischemia, perioperative hypothermia potentially increases negative postsurgery outcomes. We conducted an observational study on patients receiving elective isolated CABG to determine whether hypothermia was an independent risk factor for increased postoperative morbidity and mortality.

## Methods

Ethical approval for this study (Ethical Committee N° 201910022RINA) was provided by the Ethical Committee of National Taiwan University Hospital, Taiwan, (Chairperson Dai, Jun-Fang) on 22 October 2019. All methods were performed in accordance with the Declaration of Helsinki. The study took place in a tertiary university-affiliated teaching hospital between January 2018 and June 2019. Data were collected by examining perioperative and postoperative medical records. Our goal was to analyse the effects of perioperative core temperature in patients receiving CABG. Medical records and anaesthetic records were consecutively reviewed, and variables such as age, sex, medical history, inotropic dose, blood transfusion amounts, hemodynamic parameters, and postoperative outcomes were recorded. The follow-up period ended in October 2019. Only patients receiving elective CABG were recruited. Patients who had other procedures, such as mitral valve plasty, tricuspid valve plasty, and mitral valve replacement, were excluded. Patients with body temperatures higher than 38 °C before surgery were also excluded.

Temperatures of all patients were measured by pulmonary artery catheter and nasopharyngeal temperature after general anaesthesia till the end of surgery. Rectal temperature was measured only if cardiopulmonary bypass was used. Hypothermia was defined as blood temperature less than 36 °C measured by pulmonary artery catheter^21^. Perioperative temperature was recorded every 5 min in the anaesthetic records, and temperature upon ICU admission was documented in both ICU records and anaesthesia records. Perioperative hypothermia was defined by hypothermia duration more than 10 min. We used either forced-air system, Bair Hugger (Sterile Cardiac Access blanket Model 645; Augustine SA, Berne, Switzerland) or circulating-water blanket routinely in all our cardiac patients. If patients’ core temperature dropped below 36 °C despite above management, we would use fluid warmers to minimize heat loss. We used normothermic CPB for elective CABG patients. During CPB, the patients’ temperature was maintained between 35.5 and 36.5 °C. Our patients were separated from CPB when both the rectal and nasopharyngeal temperatures were more or equal to 36 °C. EuroSCORE II was calculated before every elective CABG surgery and documented in the medical records^22^. To compare the inotropic medication in different patients, their medication was converted to vasoactive-inotropic score (VIS)^23^. Cardiac output and systemic vascular resistance were measured using the thermodilution method. Complications included mortality, stroke, surgical site infection, prolonged ventilator use, and prolonged intensive care unit stay were retrieved from medical records. Mortality was defined by death within 30 days of the index operation.

Descriptive data are shown as means ± standard deviation for continuous variables. To analyse continuous and categorical variables, Mann–Whitney *U* tests and chi-square tests were conducted. Univariate analysis was used to screen for possible risk factors for hypothermia and prolonged ICU stay, and multivariate logistic regression model was established to estimate odds ratio and adjust for confounding factors. All statistical analyses were performed using the STATA® 13.0 MP statistical package (StataCorp LP, College Station, TX, USA). A p value of less than 0.05 was considered statistically significant.

### Ethics approval and consent to participate

Ethical approval for this study (Ethical Committee N° 201910022RINA) was provided by the Ethical Committee of National Taiwan University Hospital, Taiwan, (Chairperson Dai, Jun-Fang) on 22 October 2019. All methods were performed in accordance with the Declaration of Helsinki. Informed consent was waived by the Ethical Committee of National Taiwan University Hospital.

## Results

**Figure 1:**
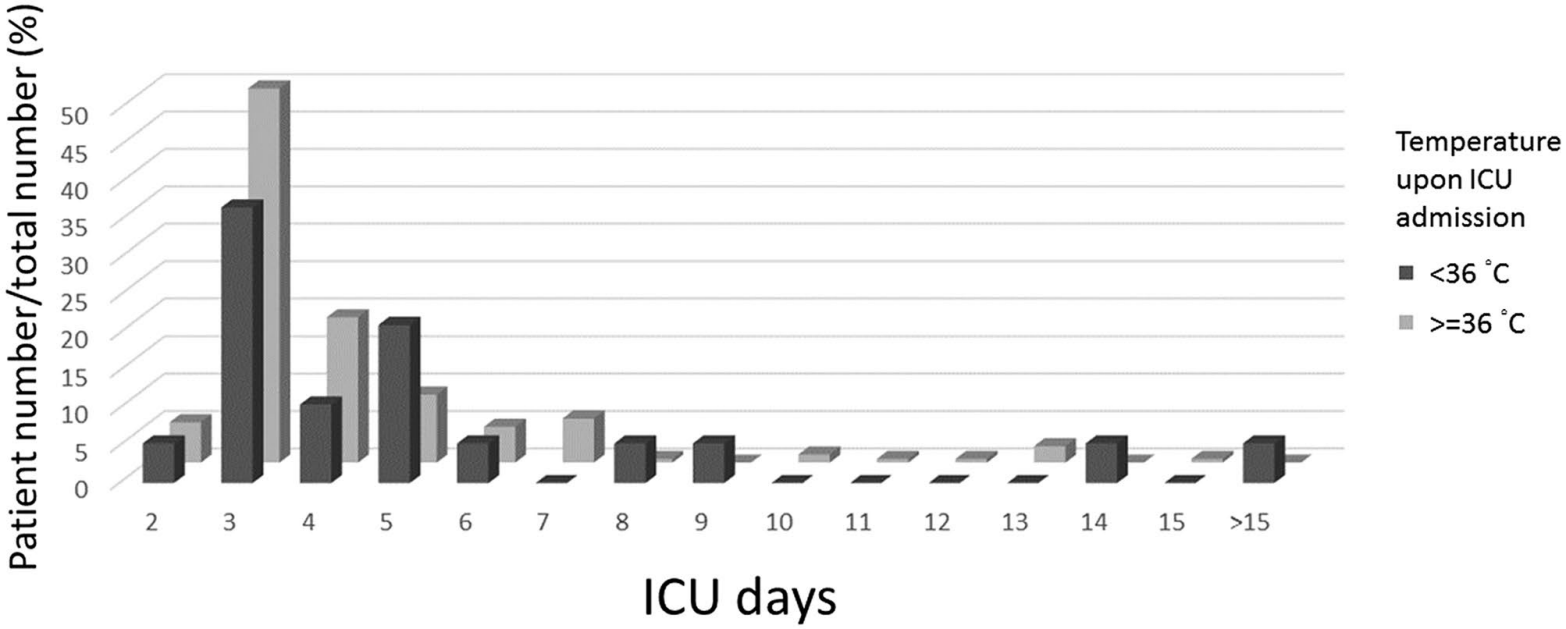
Intensive care unit stay in patients receiving elective coronary artery bypass grafting. Patients who had elective coronary artery bypass grafting are divided by temperature upon ICU admission. Their intensive care unit stays were plotted.

We included 206 patients who had elective CABG surgery in the analysis. Demographic data are presented in Table 1. No mortality or stroke was observed within 30 days postsurgery, and the SSI rate was 2.4%. For the 44 patients who were not eligible for the analysis, their EuroscoreII(%) was 8.22 ± 14.4, surgical site infection rate was 2.3%, stroke rate was 0, and 30-day mortality was 4.5%. Approximately half (56.3%) of the patients used a forced-air warming device during the surgery, and the others used circulating warm water blankets^24^. More than 60% of patients had cardiopulmonary bypass during the surgery, and the average duration of cardiac bypass was 128 min. The ICU stay distribution is displayed in Fig. 1.

**Table 1:** Baseline data in elective coronary artery bypass grafting surgery patients.

Summary of inclusion patients	
Age	64.6 ± 10.1
Sex (male %)	171 (83.0%)
Body weight (kg)	68.5 ± 13.5
Body height (cm)	164.1 ± 8.2
Body mass index (kg/m^2^)	25.4 ± 3.8
Body surface area (m^2^)	1.74 ± 0.79
Diabetes mellitus (%)	93(45.2%)
End-stage renal disease (%)	28(13.6%)
Smoking (%)	71(34.5%)
EuroScore II (%)	4.09 ± 6.37
Left ventricular ejection fraction (%)	57.0 ± 14.6
Off-pump CABG (%)	71(34.5%)
Intra-operative lowest blood temperature	35.5 ± 1.7
Temperature on ICU admission	36.3 ± 3.5
Forced air warming device use	116(56.3%)
Operation duration (min)	299.6 ± 85
Intubation time (h)	11.1 ± 24.8
ICU stay (day)	4.6 ± 5.2

Values are mean ± SD or number (proportion).

*CABG* coronary artery bypass grafting, *ICU* intensive care unit.

We divided our patients into two groups by cardiopulmonary bypass usage (on-pump or off-pump). Demographic data, medical history, and number of blood transfusions were similar in the two groups (Supplementary table). Although inotropic use, presented as VIS score, was similar in the two groups, patients with off-pump CABG received more norepinephrine during operation (p = 0.0001). Perioperative hypothermia incidence was similar between the two groups, but patients with off-pump CABG experienced more hypothermia events upon ICU admission (p = 0.04). The forced-air warming system was used more frequently in the on-pump CABG group. (p < 0.001). The operation duration and intubation duration were significantly shorter in patients receiving off-pump than on-pump CABG (p < 0.001 and p = 0.03, respectively), but no significant difference in ICU stay was observed between on-pump and off-pump groups.

We also compared CABG patients who were hypothermic before transfer to the ICU with patients who were normothermic and found that hypothermic patients were taller (168.1 ± 2.0 cm vs 163.7 ± 0.6 cm; p = 0.03), received more off-pump CABG surgeries (57.9% vs 32.1%; p < 0.05), and used more intraoperative norepinephrine (46.2 ± 9.5 vs 25.6 ± 2.8 ug; p < 0.05). Hypothermic patients had lower left ventricular ejection fraction (LVEF) (48.4 ± 3.2% vs 57.9 ± 1.1%; p = 0.007), but EuroSCORE II and VIS score before ICU admission were similar between the two groups. Although no difference in SSI rate or intubation duration was observed, patients with hypothermia had significantly longer ICU stays (8.5 ± 3.74 days vs 4.09 ± 0.16 days; p < 0.001).

We also subgrouped our patients by cardiopulmonary bypass usage and presence of hypothermia (Table 2). More patients who had off-pump CABG were hypothermic. Compared with normothermic patients, hypothermic patients in the off-pump CABG group had lower LVEF (p < 0.05) and required more intraoperative norepinephrine. In the off-pump group, average ICU stay for hypothermic patients was significantly longer than that of normothermic patients (p < 0.001). No significant difference was observed in ICU stay duration between hypothermic and normothermic patients in the on-pump CABG group.

**Table 2:** Characteristics of hypothermia and normothermia patients in Off-pump and On-pump coronary artery bypass surgery groups.

Temperature on ICU admission	Off-pump CABG		On-pump CABG	
	Hypothermia	Normothermia	Hypothermia	Normothermia
Number	11	60	8	127
Age	62.4 ± 12.1	62.9 ± 10.5	71.8 ± 9.5	64.8 ± 9.8
Sex (male %)	10 (90.9%)	53 (88.3%)	7 (87.5%)	101 (79.5%)
Body weight (kg)	169.9 ± 9.1	165.0 ± 7.5	164.3 ± 7.7	163.1 ± 8.1
Body height (cm)	72.7 ± 15.3	70.4 ± 13.5	64.6 ± 11.8	68.0 ± 12.7
Body surface area (m^2^)	1.82 ± 0.22	1.76 ± 0.18	1.71 ± 0.18	1.72 ± 0.18
Body mass index (kg/m^2^)	25 ± 3.6	25.8 ± 4.0	24.3 ± 3.7	25.5 ± 3.6
Left ventricular ejection fraction (%)	46.0 ± 15.4	59.1 ± 14.3 (p = 0.007)	54.2 ± 10.4	57.7 ± 14.1
EuroSCORE II (%)	3.76 ± 3.66	2.98 ± 4.09	8.8 ± 9.3	3.89 ± 6.76
Diabetes mellitus (%)	6 (54.5%)	25 (41.7%)	5 (57.1%)	57 (46.2%)
End-stage renal disease (%)	2 (18.2%)	8 (13.3%)	2 (28.6%)	13 (11.1%)
Smoking (%)	2 (18.2%)	18 (30%)	5 (71.4%)	46 (36.8%)
Lowest temperature in the operation theater	35.4 ± 0.3	35.9 ± 0.5 (p = 0.0015)	35.4 ± 0.9	35.8 ± 0.5
Cardiac output (L/min)	3.95 ± 1.36	3.59 ± 0.94	3.08 ± 0.83	3.33 ± 1.07
SVR after induction (dyn s/cm^5^)	1434.5 ± 540.4	1515.9 ± 509	1486.7 ± 525	1594.1 ± 621.2
VIS score	1.1 ± 2.0	2.1 ± 6.6	3.3 ± 3.7	2.0 ± 2.7
Intraoperative norepinephrine dose (ug)	63 ± 41.4	37.7 ± 47.7	22.1 ± 19.3	19.6 ± 30.5
SVR after protamine (dyn s/cm^5^)	1299.3 ± 522.4	1168.2 ± 447.8	983.7 ± 213.9	1010.5 ± 422.9
PRBC transfusion(U)	0	0.2 ± 0.5	0.8 ± 1.1	0.3 ± 0.8
Platelet transfusion (U)	6 ± 8.5	8.7 ± 10.7	4.8 ± 6.6	9.0 ± 11.0
Forced air warming system (%)	2(18.2%)	21(35%)	5(57.1%)	88(69.3%)
Operation duration (min)	227.9 ± 33.5	226.1 ± 35.8	312.3 ± 82.9	331.4 ± 70.2
Cardiopulmonary bypass duration (min)	0	0	174.3 ± 107.8	184.0 ± 95.7
Intubation time (h)	13.2 ± 18.5	4.7 ± 3.9	21.1 ± 16.2	11.6 ± 25.8
ICU stay (day)	10.5 ± 20.3	4.0 ± 2.2 (p = 0.016)	5.4 ± 2.3	4.2 ± 2.2
Surgical site infection	0	1(1.7%)	0	4(3.4%)

Values are mean ± SD or number (proportion).

*PRBC* packed red blood cell, *VIS* vasoactive inotropic score, *ICU* intensive care unit; Hypothermia defined as blood temperature < 36 C; *SVR* Systemic vascular resistance.

We conducted multivariable linear regression for hypothermia before transfer to the ICU with stepwise regression by forward selection method. After adjusting for age, sex, height, LVEF, CPB use, forced-air warming system use, and intraoperative norepinephrine dosage, we found that body height, lower LVEF, and off-pump CABG were independent risk factors for hypothermia (p = 0.012, 0.016, and 0.04, respectively). Off-pump CABG and high perioperative norepinephrine use were confounding factors for hypothermia upon ICU admission. The use of a forced-air warming device did not reduce hypothermia events.

Univariate analysis was done to screen for possible risk factors of prolonged ICU stay in CABG patients. Risk factor with a p value below 0.1 was included in the multivariate logistic regression model (Table 3). We chose basic characteristics (sex, age), LVEF, and EuroSCORE II, and hypothermia upon ICU arrival into our model, and found that higher EuroSCORE II (p < 0.001; odds ratio 1.2) and hypothermia before transfer to the ICU (p = 0.04; odds ratio 3.8) were independent risk factors for prolonged ICU stay.

**Table 3:** Univariate analysis for risk factors affecting ICU stay in CABG patients.

	Univariate analysis		Multivariate analysis	
	Correlation coefficient	p-value	Odds ratio	p-value
Age	0.04	0.53	1.0	0.26
Sex	− 0.05	0.51	1.5	0.70
Body weight (kg)	− 0.05	0.51		
Body height (cm)	0.09	0.18		
Diabetes mellitus (%)	0.07	0.29		
End-stage renal disease (%)	0.07	0.31		
Smoking (%)	− 0.06	0.41		
EuroscoreII (%)	0.33	< 0.001	1.2	< 0.001
Left ventricular ejection fraction (%)	− 0.25	0.0003	1.0	0.12
Cardiac output (L/min)	− 0.02	0.77		
Systemic vascular resistance (dyn s/cm^5^)	0.10	0.17		
Pulmonary vascular resistance (dyn s/cm^5^)	0.08	0.32		
Cardiopulmonary bypass use	0.06	0.41		
Intra-operative lowest blood temperature	0.03	0.65		
Temperature on ICU admission	0.23	0.0007	3.8	0.04

*ICU* intensive care unit, *CABG* coronary artery bypass grafting.

## Discussion

Our study showed that hypothermia before transfer to the ICU was an independent risk factor for prolonged ICU stay. This finding was especially significant in off-pump CABG patients. Hypothermia did not influence intubation duration or SSI.

There was no incidence of in-hospital mortality in our study. Therefore, we cannot discuss whether hypothermia was associated with increased mortality. Furthermore, hypothermia was not associated with increased blood transfusion. Preoperative consultation of anticoagulant adjustment, use of absorbable haemostatic materials, and advanced surgical techniques may have decreased the need for blood transfusion, making hypothermia a less important factor for blood transfusion in our analysis. SSI rate was fairly low in our study and was not related to hypothermia. A previous clean orthopaedic study also revealed that hypothermia was not correlated with higher SSI rate.^10^ It is possible that in elective clean surgeries, hypothermia is not an important factor for SSI. Though moderate intraoperative hypothermia was known to promote myocardial injury, surgical site infections, and blood loss, aggressive warming did not decrease major cardiovascular outcomes non non-cardiac surgical patients when compared to routine care group (35·5 °C) in randomized controlled trial^25^. Our study showed that elective cardiac patients had similar results.

We did not follow-up the core temperature of our patients in the ICU because the temperature recording was not unified. During the operation, we used PA thermistor to measure central blood temperature because it is the gold standard for core temperature measurement^26^. Nasopharyngeal temperature is routinely monitored in cardiac surgery in our institution, and the measurements correlated well with PA catheter. However, the measurement would be interfered if the patient had much secretion. We also monitored rectal temperature, but these readings may be affected by the presence of stool and of bacteria that generate heat^27^. In ICU, PA catheter was removed according to the disease status, thus not all patients had detailed core temperature data. Furthermore, the temperature was not continuously monitored. We could collect temperature data every 10 min from anesthetic record, but the data was once every 1 to 4 h in ICU record. The ANZICS registry study showed that nearly half of all non-cardiac patients experienced hypothermia in the first 24 h in ICU, but hypothermia resolved within 3 h of ICU admission in more than 80% of patients. Furthermore, hypothermia was not independently associated with increased hospital mortality^28^. Thus we focused on temperature management in perioperative care.

We found that lower LVEF was an independent risk factor for hypothermia, which implies that hypothermia may be a marker of illness severity. The warming process after hypothermia could increase oxygen demand and lead to negative outcomes. Compared with on-pump CABG, patients receiving off-pump CABG experienced greater drops in body temperature postsurgery, and significantly more were hypothermic upon ICU admission despite the use of a forced-air warming device or circulating warm water blankets (Supplementary Table). CPB with a heat exchanger may be better able to maintain and sustain a target temperature for a longer duration. Previous study demonstrated that active warming may reduce morbidity^29^. Thus, in off-pump CABG surgery, room temperature should be adjusted accordingly^30^, and patient-warming devices should be applied for temperature management.

A major strength of our study is that it included only elective coronary bypass patients in a single-centre site that conducts a large number of cardiovascular surgeries. This eliminated the potential bias in patient selection, CPB management, and clinical care. In addition, we were able to obtain detailed anaesthetic records, hemodynamic monitors, inotropic use, and precise blood temperature records during the operation, which may not be feasible in a large database study^18^. The major limitation of our study is that it was observational and therefore could only demonstrate associations and not causations. In addition, our study was not large enough to detect weak associations, especially when the event incidence was low. Nevertheless, after adjustment for EuroSCORE II, hypothermia upon ICU admission was significantly associated with longer ICU stay. Although we could not derive a causal relationship between hypothermia and longer ICU stay due to the retrospective study design, our findings suggest that further studies, including randomized controlled trials, may be useful to determine if correcting hypothermia improves clinical outcomes.

## Conclusions

Hypothermia upon ICU admission was an independent risk factor for prolonged ICU stay in patients receiving elective coronary artery bypass surgery.

## Supplementary Information

Supplementary Table S1.

## Data Availability

The datasets used and analysed during the current study available from the corresponding author on reasonable request.

## Abbreviations

CABG: Coronary artery bypass grafting
ICU: Intensive care unit
SSI: Surgical site infection
VIS: Vasoactive-inotropic score
LVEF: Left ventricular ejection fraction
PRBC: Packed red blood cell
