# Supplementary material for: Hypothermia is an independent risk factor for prolonged ICU stay in coronary artery bypass surgery: an observational study
Source: Sci Rep. 2023 Mar 21;13:4626. doi: 10.1038/s41598-023-31889-x (PMC10030842; doi:10.1038/s41598-023-31889-x)
Supplement: Supplementary file 1 — Supplementary Table S1. [file 41598_2023_31889_MOESM1_ESM.docx]

Supplementary Table. Characteristics of Off-pump and On-pump coronary artery bypass surgery patients. Values are mean±SD or number (proportion).

|  | Off-pump CABG | On-pump CABG | p-value |
| --- | --- | --- | --- |
| Number(%) | 71(34.5%) | 135(65.5%) |  |
| Age | 62.8±1.3 | 65.5±0.8 | 0.07 |
| Sex(male %) | 63(88.7%) | 108(80.0%) | 0.11 |
| Body weight(kg) | 70.7±1.6 | 67.3±1.1 | 0.07 |
| Body height(cm) | 165.7±0.9 | 163.2±0.7 | 0.02 |
| Body mass index(kg/m^2^) | 25.6±0.5 | 25.2±0.3 | 0.46 |
| Body surface area(m^2^) | 1.77±0.02 | 1.72±0.17 | 0.05 |
| EuroscoreII(%) | 3.10±0.5 | 4.6±0.6 | 0.05 |
| Left ventricular ejection fraction (%) | 57.1±1.8 | 56.9±1.3 | 0.80 |
| Cardiac output(L/min) | 3.6±1.0 | 3.3±1.1 | 0.06 |
| Systemic vascular resistance (dyn·s·cm^−5^) | 1505.4±509 | 1588.6±615 | 0.36 |
| Diabetes mellitus(%) | 31(43.7%) | 62(45.9%) | 0.77 |
| End-stage renal disease (%) | 10(14.1%) | 18(13.3%) | 1.00 |
| Smoking (%) | 20(28.2%) | 51(37.8%) | 0.2 |
| VIS score | 1.98±6.1 | 2.09±2.7 | 0.86 |
| Intra-operative norepinephrine dose (ug) | 41.3±5.7 | 19.8±2.6 | 0.0001 |
| PRBC transfusion(U) | 0.2±0.1 | 0.3±0.08 | 0.45 |
| Platelet transfusion (U) | 8.4±2.3 | 8.8±1.1 | 0.88 |
| Hypothermia during operation | 39(54.9%) | 77(57.0%) | 0.88 |
| Hypothermia on ICU admission | 11(15.5%) | 8(5.9%) | 0.04 |
| Forced air warming system use | 23(32.4%) | 93(68.9%) | <0.001 |
| Operation duration(minutes) | 226.4±4.2 | 338.1±6.7 | <0.001 |
| Intubation time(hours) | 6.1±1.0 | 13.8±2.6 | 0.03 |
| ICU stay(days) | 4.97±0.98 | 4.35±0.19 | 0.08 |

PRBC: packed red blood cell; VIS: vasoactive inotropic score; ICU: intensive care unit; Hypothermia defined as blood temperature < 36 C
